# Retroperitoneal Castleman’s disease: advocating a multidisciplinary approach for a rare clinical entity

**DOI:** 10.1186/1477-7819-12-30

**Published:** 2014-02-04

**Authors:** Austin D Williams, Adriana Sanchez, Jun Steve Hou, Rene Rothstein Rubin, Mark E Hysell, Blake D Babcock, Mohammad F Shaikh, Michael S Weingarten, Wilbur B Bowne

**Affiliations:** 1 Division of Surgical Oncology, Department of Surgery, Drexel University College of Medicine, 254 N. 15th St., MS 413, Philadelphia, PA 19102, USA; 2 Department of Pathology, Drexel University College of Medicine, 254 N. 15th St., MS 435, Philadelphia, PA 19102, USA; 3 Division of Hematology/Oncology, Department of Internal Medicine, Drexel University College of Medicine, 245 N. 15th St., 8th Floor, Philadelphia, PA 19102, USA; 4 Department of Diagnostic Radiology, Hahnemann University Hospital, 230 N. Broad St., MS 206, Philadelphia, PA 19102, USA; 5 Division of Vascular Surgery, Department of Surgery, Drexel University College of Medicine, 254 N. 15th St., MS 413, Philadelphia, PA 19102, USA

**Keywords:** Angiofollicular lymph node hyperplasia, Castleman’s disease, Management, Multidisciplinary care

## Abstract

**Background:**

Castleman’s disease is a rare and poorly understood disease entity that may resemble more common conditions and represents a clinical challenge to the treating surgeon.

**Case presentation:**

In this report, we describe a case of a 61-year-old Caucasian woman with a symptomatic retroperitoneal mass. The specimen obtained from her resection contained a protuberant encapsulated mass, exhibiting microscopic features consistent with localized, unicentric Castleman’s disease. These characteristics included architectural features and immunohistochemical findings consistent with the hyaline vascular variant of Castleman’s disease.

**Conclusion:**

We report a very rare case of a retroperitoneal hyaline vascular type of Castleman’s disease. We discuss the diagnostic dilemma Castleman’s disease may present to the surgeon, with an emphasis on multidisciplinary management of these patients. We also review current data on pathogenesis, treatment and outcomes.

## Background

Angiofollicular lymph node hyperplasia, or Castleman’s disease (CD), is a rare neoplastic disease first identified in 1956 by Castleman and colleagues [1]. It has been described mainly in case reports and small series, thus the incidence and prevalence of CD are difficult to ascertain. CD is characterized by massive growth of nonclonal lymphoid tissue and has been categorized according to anatomic location, histological presentation and centricity (local vs. multicentric). In 65% to 80% of cases, CD is primarily found in the mediastinum, with an increasing incidence noted in the head and neck region. Mesenteric, retroperitoneal and pelvic tumors are unique and discovered less frequently [2-4]. Clinical imaging cannot readily distinguish CD from other neoplastic diseases. Published reports have described CD mimicking lymphoma, carcinoma and sarcoma in various anatomic sites, such as the pancreas, liver and spinal cord [5-7].

The classic histological subtype of CD, representing 76% to 91% of localized disease, is the hyaline-vascular type (HV-CD). The plasma cell variant (PC-CD) has been identified in 9% to 24% of CD cases, and a rare hyaline-vascular plasma cell (or mixed) subtype has also been reported [2,8,9]. Clinically, HV-CD is most often found in indolent unicentric CD tumors (UCDs), and a more generalized (multicentric) lymphadenopathy, which frequently presents with constitutional symptoms, is associated with PC-CD [2,10].

CD has an unclear etiology, although human herpes virus 8 (HHV-8) and HIV coinfection has been implicated in the etiology of multicentric disease (MCD). Although it has been hypothesized that treatment with highly active antiretroviral therapies might decrease the incidence of MCD, MCD has actually increased and further study elucidating the effects of viral seropositivity on dysregulation of the immune system is ongoing [11]. Additionally, the roles of autoimmune diseases and Epstein-Barr virus (EBV) infection in the pathogenesis of CD are now being explored [12,13].

Historically, the treatment and prognosis of CD are based on the centricity of the disease rather than on the histopathology. Surgical resection of UCDs can be curative, with long-term, recurrence-free survival described in the majority of case reports and series. The tumor’s location and relationship with adjacent structures are important factors in predicting surgical morbidity [2,14]. MCD, however, often presents additional diagnostic uncertainty and treatment challenges. Immunotherapy, chemotherapy and antiviral therapy are the mainstays of treatment. Surgical treatment is reserved for either excisional biopsy, debulking of a dominant disease focus or palliative resection [14,15]. Transformation into non-Hodgkin’s lymphoma may occur, and, despite treatment, mortality due to MCD occurs in 40% of patients within 10 years from the time of diagnosis [14].

In the present report, we describe a rare case of retroperitoneal UCD and discuss a multidisciplinary approach to diagnosis and treatment of patients with this rare clinical entity.

## Case presentation

**Figure 1:**
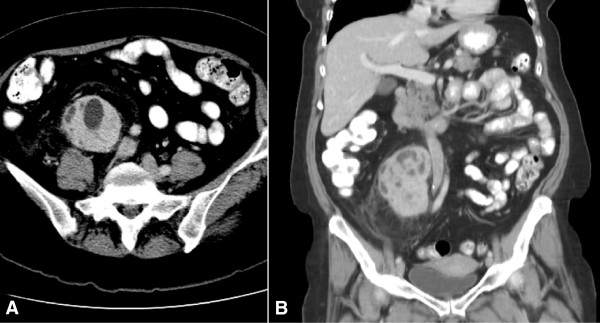
**Preoperative imaging. (A)** and **(B)** Axial and coronal computed tomographic scans, respectively, with oral and intravenous contrast showing a large, heterogeneously enhancing retroperitoneal mass deforming the inferior vena cava at the level of the right renal vein (arrow).

A 61-year-old Caucasian woman was seen by her primary care physician for a palpable abdominal mass that had been present for 1½ years. She did not complain of systemic symptoms, general malaise, weight loss, flank pain or urinary symptoms, but described mild diarrhea and right periumbilical pain that radiated to the right lower quadrant. Her medical history included well-controlled hypertension and type 2 diabetes mellitus. Her previous colonoscopic and endoscopic evaluations were normal. During her physical examination, a nontender, immobile mass was palpable in the right lower quadrant without peripheral lymphadenopathy. Examination and laboratory studies were otherwise unremarkable. Computed tomography (CT) with intravenous and oral contrast of the abdomen and pelvis revealed a heterogeneously enhancing, 10.2 × 6.9 × 6.3–cm solid mass in the right retroperitoneal space immediately lateral to and displacing the inferior vena cava and right iliac vasculature (Figure 1A and 1B). Significant deformation of adjacent retroperitoneal structures was also observed. There was no evidence of abdominopelvic adenopathy or intraperitoneal disease. A biopsy was not performed.

An exploratory laparotomy revealed a large mass with remarkable tumor and perivascular inflammatory changes extending to the right iliac vessels and over the inferior vena cava. The mass was completely resected without the need for multiorgan resection. The blood loss during surgery was approximately 1 L. The patient underwent a complete curative resection. She recovered well following the operation and was discharged on the seventh postoperative day. The patient has remained without radiologic evidence of disease recurrence during 2 years of follow-up.

### Pathological findings

**Figure 2:**
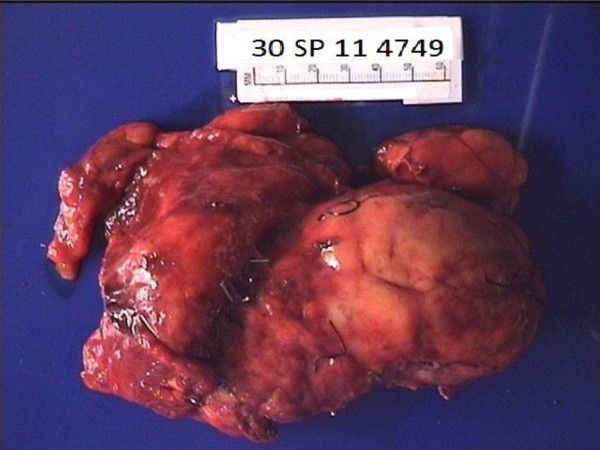
**Gross specimen.** The resected specimen surfaces weighed 340 g and measured 18 × 11 × 7 cm. The tissue specimen contained an encapsulated mass that measured 11.5 × 6.0 × 5.0 cm and a medial border 2.0 cm from the border of the inferior vena cava.

A soft encapsulated mass measuring 11.5 × 6.0 × 5.0 cm with tannish orange cut surfaces was identified in a surgical tissue specimen that had attached fatty and inflammatory soft tissue (Figure 2). All surgical margins were clear, with the closest approximately 2.0 cm from the lateral aspect of the inferior vena cava.

**Figure 3:**
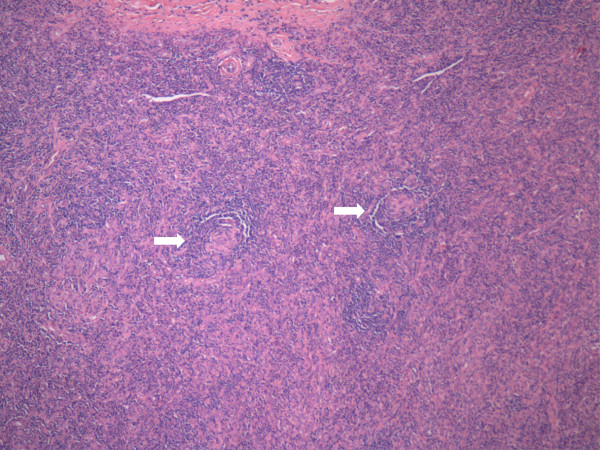
**Histopathology.** Microscopy of resected section showing two atrophic, hyalinized germinal centers (two-headed arrow) (hematoxylin and eosin stain; original magnification 4×).

**Figure 4:**
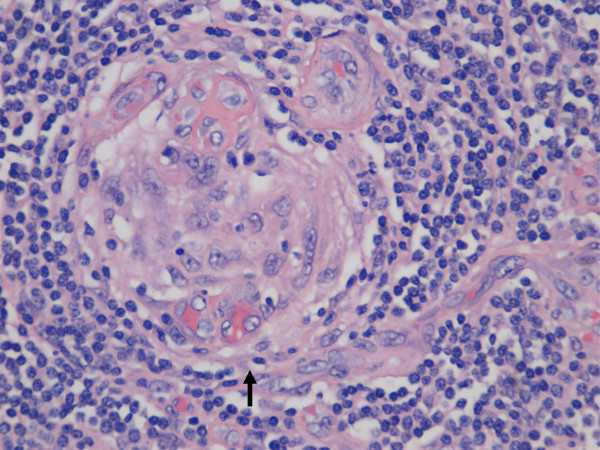
**Histopathology.** Microscopy of a section taken from the mass exhibiting the hyaline vascular features of Castleman’s disease, including a characteristic endothelium-lined blood vessel radially penetrating an atrophic germinal center (arrow) (hematoxylin and eosin stain; original magnification, 20×).

**Figure 5:**
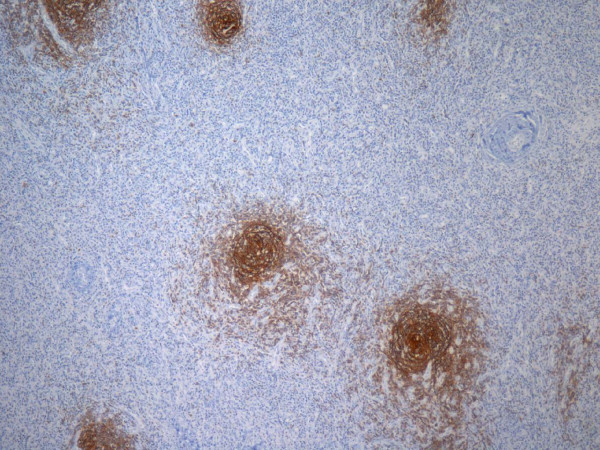
**Histological image with immunostaining for cluster of differentiation 21 antibody.** highlights the follicular dendritic network of residual germinal centers (arrow) (original magnification, 4×).

Under the microscope, we could see that the nodule consisted of lymphocytes with marked vascular proliferation and prominent hyalinization. Low- and high-power histopathology revealed regressively transformed germinal centers with normal B-cell distribution surrounded by hypervascular mantle zones composed predominantly of concentric rings (“onion-skinning”) of small cluster of differentiation 4 (CD4)-positive T lymphocytes and plasma cells (Figures 3 and 4). We frequently observed hyalinized blood vessels perforating the follicles from the mantle zone. Immunostaining for CD21 demonstrated the characteristic follicular dendritic network of residual germinal centers (Figure 5). CD10 was also weakly positive in some of the transformed germinal centers. PCR analysis of paraffin-embedded tissue targeting the T-cell receptor γ gene revealed no gene rearrangement. *In situ* hybridization for EBV and immunostaining for HHV-8 were both negative. These architectural features and immunochemical findings are consistent with HV-CD.

## Discussion

Our patient presented to our institution with a rare, symptomatic, retroperitoneal, unicentric HV-CD tumor. Although etiologic factors continue to be elucidated [7], a major clinical challenge rests first on establishing the diagnosis of CD. An important initial step is a high clinical index of suspicion for CD in symptomatic patients [2]. Regardless of whether it presents as an incidental finding or a symptomatic mass, CD is rarely included within differential diagnoses. Multidisciplinary review is highly recommended and may provide important insight into establishing the diagnosis of CD.

Primary retroperitoneal tumors are particularly difficult to diagnose because of their location and often are clinically detected only after the tumor reaches a size that is large enough to be found with a resultant mass effect and involvement of surrounding structures. Among primary retroperitoneal tumors, one-third are sarcomas [16]. Liposarcomas comprise 70% of retroperitoneal sarcomas and can often be differentiated on cross-sectional imaging on the basis of fat distribution [17]. Lymphomas, carcinomas and neuroendocrine tumors, as well as extragonadal germ cell tumors, and metastatic melanomas, also occur in the retroperitoneum

The decision to biopsy retroperitoneal masses preoperatively remains controversial. At our institution, if a mass is suspected to be malignant and appears amenable to complete surgical resection, we frequently recommend surgical management without preoperative biopsy. Most surgeons agree that surgical resection in the appropriate clinical setting without biopsy is acceptable [18]. Biopsy is useful, however, in cases where radiology is inconclusive or tissue diagnosis prior to surgery may alter subsequent management [19]. Biopsy is also reasonable in patients whose performance status may preclude safe surgical resection, thus necessitating alternative therapies or treatment approaches. CT- or ultrasound-guided percutaneous biopsy is frequently used, and biopsy by endoscopic ultrasound has proven efficacious for retroperitoneal masses adjacent to the bowel [20]. Core-needle biopsy is often preferable to fine-needle aspiration to obtain appropriate specimens for pathologic evaluation, to differentiate variant histologic subtypes of mesenchymal tumors and to distinguish other types of tumors found within the retroperitoneum [21]. Furthermore, evaluation of rare clinical entities such as CD requires histologic architectural preservation provided by substantial tissue biopsies to render an accurate diagnosis.

An important clinical consideration in CD tumors is the degree of vascularity, which is more prominent in the HV-CD variant than in the PC-CD type. The degree of vascularity is relevant to both the diagnosis and treatment of CD. When CD presents in a rare location (for example, the head of the pancreas), the increased vascularity may confound the diagnosis and cause suspicion for primary malignancy with associated angiogenesis [22]. Zheng *et al.*[23] described characteristic findings of CD on CT scans, such as the degree of rim enhancement, that can assist in distinguishing CD from primary malignancy. Care must also be taken to differentiate UCD from MCD by using radiologic techniques, as treatment for these variants differs significantly. At our institution, we routinely employ dedicated 256 × 256 matrix multidetector CT cross-sectional imaging with arterial and venous phases for diagnostic and preoperative planning. Surgical resection of highly vascular tumors is technically challenging and is associated with morbidity and possible mortality related to blood loss. Recently published case reports have described the utilization of preoperative angiography and embolization to decrease intraoperative bleeding and subsequent morbidity [24-26].

Once the diagnosis or real clinical suspicion of UCD has been established, complete surgical resection is the treatment of choice on the basis of its reported long-term, recurrence-free survival rate with possible cure [2,14]. Importantly, surgical resection of large vascular tumors may be technically challenging, especially in the mediastinum and retroperitoneum, where vital structures tend to be involved with the tumor itself or with associated surrounding inflammatory and/or desmoplastic disease response. In many cases, resultant compression of vital structures due to mass effects necessitates the need to remove these lesions. Novel neoadjuvant approaches to facilitate safe surgical resection have been proposed. Bandera *et al*. [27] reported the successful use of rituximab (an anti-CD20 monoclonal antibody) in the neoadjuvant setting of mediastinal UCD involving the pulmonary artery and superior vena cava, followed by successful surgical resection. de Vries *et al*. [28] described the efficacious use of neoadjuvant radiotherapy for an unresectable abdominal UCD involving the iliac vessels to downsize the tumor, followed by complete surgical resection. In addition, a limited number of reports have addressed the response of CD to radiotherapy (2,700 to 4,500 cGy) administered to involved sites, with resultant remission of disease in isolated cases, suggesting a possible role for radiotherapy in a neoadjuvant or adjuvant setting [29-32]. Our present case report demonstrates the association of a CD-HV-type tumor with the inferior vena cava and in close proximity to the iliac vessels, which we treated with a technically challenging complete surgical resection. More investigation is required to further evaluate the utility of neoadjuvant strategies with the potential to reduce the size of tumors in cases where surgery may present a significant risk to the patient. In certain cases, if complete resection is not possible, partial resection or observation with long-term follow-up may be useful. Appropriate follow-up after surgical resection for the CD-HV type has not been established. We recommend routine CT yearly for the first 3 years and again at 5 years postoperatively. Thereafter follow-up imaging should be clinically driven based upon suspicion for disease recurrence.

Although the decision-making regarding of UCD and its management is challenging, MCD presents an even greater problem for the treating physician and surgeon. The use of antiviral and antiretroviral therapies is considered necessary with the addition of chemotherapeutic agents, including regimens developed for treatment of lymphoma and rituximab alone, which have demonstrated variable responses [15]. Despite aggressive medical management, the rates of relapse, malignant transformation and disease-related mortality remain high.

## Conclusion

In this case report, we describe a rare case of retroperitoneal unicentric HV-CD, a poorly understood disease that creates a diagnostic and therapeutic dilemma for surgeons. To date, the use of improved radiologic criteria for diagnosis, interventional techniques and operative, neoadjuvant and adjuvant strategies appear to play an increasingly important role in the diagnosis and management of patients with this rare disease. Because of the rarity of CD, the opportunity for clinical trials to produce uniform evidence-based approaches is unlikely. With regard to the treatment options for patients with these rare tumors, we encourage a collaborative, multidisciplinary approach that includes surgical specialists, radiologists, pathologists and oncologists to discuss treatment options to optimize patient outcomes.

## Consent

Written informed consent was obtained from the patient for publication of this case report and any accompanying images. A copy of the written consent is available for review by the Editor-in-Chief of this journal.

## Competing interests

The authors declare that they have no competing interests.

## Authors’ contributions

AW acquired data and drafted and revised the manuscript. AS and SH made the pathologic diagnosis and revised the manuscript. RR, MH, BB and MS were the pre- and postoperative treatment team and revised the manuscript. MW was responsible for surgical management and revised the manuscript. WB was the treating surgeon and drafted and revised the manuscript. All authors read and approved the final manuscript.
